# Efficacy of an mHealth App to Support Patients’ Self-Management of Hypertension: Randomized Controlled Trial

**DOI:** 10.2196/43809

**Published:** 2023-12-19

**Authors:** Fang Liu, Ting Song, Ping Yu, Ning Deng, Yingping Guan, Yang Yang, Yuanji Ma

**Affiliations:** 1 Health Management Center General Hospital of Ningxia Medical University Yinchuan China; 2 Department of Cardiology General Hospital of Ningxia Medical University Yinchuan China; 3 Centre for Digital Transformation, School of Computing and Information Technology, Faculty of Engineering and Information Sciences University of Wollongong Wollongong Australia; 4 The Ministry of Education Key Laboratory of Biomedical Engineering, College of Biomedical Engineering and Instrument Science Zhejiang University Hangzhou China; 5 Department of Cardiology Zhongshan Hospital, Fudan University Shanghai China; 6 Shanghai Institute Of Cardiovascular Diseases Shanghai China; 7 National Clinical Research Center for International Medicine Shanghai China

**Keywords:** mobile app, mHealth, mobile health, smartphone, high blood pressure, medication adherence, reminder, health education, motivation, hypertension control, hypertension, blood pressure, self-management

## Abstract

**Background:**

Hypertension is a significant global disease burden. Mobile health (mHealth) offers a promising means to provide patients with hypertension with easy access to health care services. Yet, its efficacy needs to be validated, especially in lower-income areas with a high-salt diet.

**Objective:**

This study aims to assess the efficacy of an mHealth app–based intervention in supporting patients’ self-management of hypertension.

**Methods:**

A 2-arm randomized controlled trial was conducted among 297 patients with hypertension at the General Hospital of Ningxia Medical University, Ningxia Hui Autonomous Region, China. Participants selected via convenience sampling were randomly allocated into intervention and control groups. Intervention group participants were trained and asked to use an mHealth app named Blood Pressure Assistant for 6 months. They could use the app to record and upload vital signs, access educational materials, and receive self-management reminders and feedback from health care providers based on the analysis of the uploaded data. Control group participants received usual care. Blood pressure (BP) and 2 questionnaire surveys about hypertension knowledge and lifestyle behavior were used to assess all participants at baseline and 6 months. Data analysis was performed with SPSS software using 2-tailed *t* tests and a chi-square test.

**Results:**

There were no significant differences in baseline characteristics and medication use between the 2 groups (all *P*>.05). After 6 months, although both groups show a significant pre-post improvement (*P*<.001 each), the BP control rate (ie, the proportion of patients with a systolic BP of <140 mm Hg and diastolic BP of <90 mm Hg) in the intervention group was better than that in the control group (100/111, 90.1% vs 75/115, 65.2%; *P*<.001). The mean systolic and diastolic BP were significantly reduced by 25.83 (SD 8.99) and 14.28 (SD 3.74) mm Hg in the intervention group (*P*<.001) and by 21.83 (SD 6.86) and 8.87 (SD 4.22) mm Hg in the control group (*P*<.001), respectively. The differences in systolic and diastolic BP between the 2 groups were significant (*P*<.001 and *P*=.01, respectively). Hypertension knowledge significantly improved only in the intervention group in both pre-post and intergroup comparisons (both *P*<.001). However, only intragroup improvement was observed for lifestyle behaviors in the intervention group (*P*<.001), including medication adherence (*P*<.001), healthy diet (*P*=.02), low salt intake (*P*<.001), and physical exercises (*P*=.02), and no significant difference was observed in the control group or on intergroup comparisons.

**Conclusions:**

This research shows that the mHealth app–based intervention has the potential to improve patient health knowledge and support self-management among them toward a healthier lifestyle, including medication adherence, low-salt diets, and physical exercises, thereby achieving optimal BP control. Further research is still needed to verify the specific effects of these interventions.

**Trial Registration:**

Chinese Clinical Trial Registry ChiCTR1900026437; https://www.chictr.org.cn/showproj.html?proj=38801

## Introduction

Hypertension is a global public health concern, affecting 26% of the world’s population, with its prevalence projected to increase to 29% by 2025 [1]. Each year, hypertension is responsible for 8.5 million deaths from cardio-cerebral vascular diseases and chronic renal diseases worldwide, estimated to cost US $370 billion annually, or 10% of health care expenditure [2,3]. Previous studies have shown that even a modest reduction in blood pressure (BP) can reduce the risk of hypertension-associated morbidity and premature mortality [4,5].

Despite recognizing the harm of hypertension and the efficacy of traditional hypertension treatment approaches, hypertension control remains suboptimal for most patients, even in high-income countries [6,7]. In China, 270 million people experience hypertension; however, more than 46.3% of them are unaware of their conditions, and only 13.8% have adequately controlled hypertension [8,9]. One of the main reasons is a lack of adherence to antihypertensive medications (the mainstay of hypertension treatment) and healthy lifestyle behaviors, thus necessitating patient self-management strategies that follow various hypertension management guidelines [9-13].

In China, most patients with hypertension seek treatment in larger urban hospitals rather than local primary care clinics, as the hierarchical health care system is not as strict as that in some other countries, and patients have more freedom to choose where to receive treatment. This has led to inadequate communication between health care providers and patients about home-based self-management strategies [14-18]. Other barriers to patients’ self-management of hypertension include the lack of self-management knowledge and skills [19,20], unawareness or neglect of follow-up care [19], forgetfulness [20-22], the lack of motivation to take medications on time [20,23], and misunderstanding of prescription instructions or side effects [19,20,24]. Therefore, innovative methods, tools, and processes need to be developed to address these issues.

With the proliferation of the internet and the penetration of mobile technologies to all walks of life, we are living in a time with the most favorable conditions for leveraging our mobile devices at hand to support our health and well-being [25,26]. Mobile health (mHealth) refers to the use of mobile devices, such as mobile phones, personal digital assistants, or other wireless devices, to deliver medical or public health services [27-29]. With more than 5 billion people worldwide owning mobile phones and having access to the internet as of 2022 [30], mHealth is increasingly considered by decision makers as a potential low-cost solution to automatically link community-dwelling patients with their health care providers and to innovate hypertension outpatient services to address the abovementioned hypertension self-management challenges [31,32].

Despite numerous mHealth apps having entered the market to support patients’ self-management of hypertension, the quality of these apps varies largely [33-36]. Although some apps were well-designed and can support patients’ self-management of hypertension [37-42], overall, the efficacy of mHealth has not been established due to the heterogeneity of studies in terms of a theoretical basis, intervention design, implementation process and duration, and patient characteristics [39,40,43-45]. Moreover, the existent empirical research on the role of mHealth in supporting patients’ self-management of hypertension has been mainly conducted in Western countries [46-51]. To date, there is little research on the efficacy of mHealth services in supporting patients’ self-management of hypertension in China, especially in lower-income areas with a high-salt diet, for example, the Ningxia Hui Autonomous Region.

China faces enormous challenges in hypertension management, including the large population base, regional differences, numerous ethnic minority groups, dietary differences, the disparity in medical resource allocation, and the population’s lack of sufficient knowledge about hypertension [9,13,34]. For example, patients with hypertension often do not monitor their BP, likely due to a lack of awareness of the disease or a perceived burden to measure BP at home [20,52]. Therefore, this study aims to compare the efficacy of an mHealth app–based intervention with usual care in supporting patients’ self-management of hypertension. 

## Methods

### Ethical Considerations

This study was approved by the Human Research Ethics Committee of the General Hospital of Ningxia Medical University (ID2018-325). All participants provided formal written informed consent, which the committee approved. All names were replaced with codes during electronic data entry to preserve the anonymity and privacy of the participants. The questionnaires were locked in a filing cabinet at the trial hospital. The data were analyzed in an Excel (Microsoft Corp) spreadsheet and stored in a password-protected secure office computer of a researcher. Data security was guarded by firewalls and other security mechanisms imposed by the networked computers at the trial hospital. There was no compensation for study participation.

### Trial Design

A 2-arm, parallel, prospective randomized controlled trial (retrospectively registered in the Chinese Clinical Trial Registry: ChiCTR1900026437) was conducted to assess the 6-month effects of an mHealth app–based intervention for patients’ self-management of hypertension. The intervention was compared with usual care (control) measures at baseline and 6 months after implementation. The study followed the guidelines for reporting parallel group randomized controlled trials [53].

### Study Setting, Participants, and Recruitment

Participants were recruited from April 2017 through January 2019 at the General Hospital of Ningxia Medical University, Yinchuan, China—a unique tertiary hospital in the province. Inclusion criteria were (1) being aged 18 to 80 years; (2) being diagnosed with primary hypertension, defined as a systolic BP (SBP) of ≥140 mm Hg or diastolic BP (DBP) of ≥90 mm Hg [13]; (3) receiving antihypertensive medication treatment for over 1 month; and (4) owning a smartphone and being able to use it. Exclusion criteria were (1) being diagnosed with secondary hypertension, (2) being unable to express their perceptions because of mental disabilities or an inability to speak, and (3) not having a sphygmomanometer at home. Potential participants were contacted by a registered nurse during their clinic visit. After each person’s visit, the registered nurse talked with them for about 5 minutes, informed them of the purpose and detailed procedure of the study, and sought their consent to participate.

### Intervention

The mHealth app–based intervention contains three components: (1) a mobile app named Blood Pressure Assistant, (2) nurse training on the use of the app, and (3) an accredited “health manager” team that follows up with the patient users.

Blood Pressure Assistant was codeveloped by the General Hospital of Ningxia Medical University and Zhejiang University to support patients’ self-management of hypertension in Ningxia, China, where there is a high prevalence of hypertension due to a high-salt diet [54]. The development of the app was based on the social-cognitive theory, the user acceptance model, and goal-directed design [37,55].

**Figure 1:**
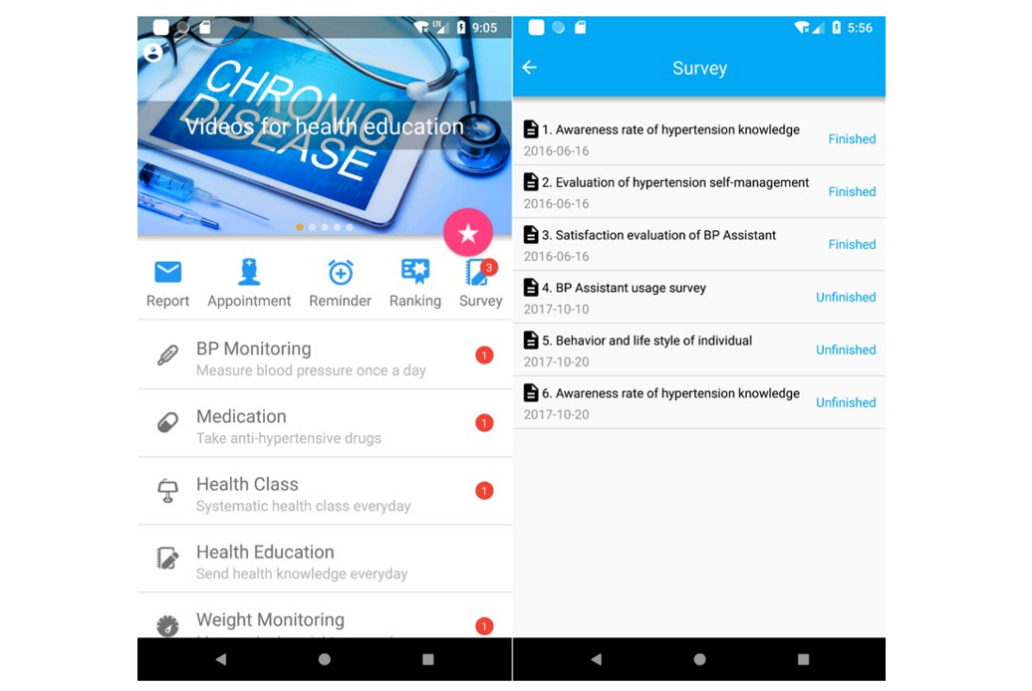
Functional modules in Blood Pressure Assistant.

Patients could self-manage hypertension using the 6 interactive functional modules in Blood Pressure Assistant: health education, health management plan, health checkup, health report, reminders to perform self-management behaviors, and performance ranking. Patients recorded BP readings and other self-management data in the app, including BP and heart rate, medication type and dose, weight, diet, salt intake, physical exercise, and uncomfortable symptoms. Digital questionnaire surveys were also available to assess patient awareness of hypertension, self-management behaviors, and user satisfaction (Figure 1). Details of the app’s functions are reported in previous studies [20,37]. There was also a web-based portal for health care providers to monitor and communicate with their patients. All uploaded data from the app can be transferred to the web portal as dynamic electronic health records. An accredited “health manager” team, including a senior medical specialist and a senior nurse, were responsible for analyzing these records; monitoring patient conditions; and providing feedback via phone calls, SMS text messages, emails, or social media (eg, WeChat) when deemed necessary.

### Sample Size

The sample size was determined through a statistical power analysis based on the change in systolic BP before and after 6 months, as it was the most critical indicator of a patient’s cardiovascular risk profile [56]. We considered a reduction in SBP of 20 mm Hg and at least 5 (SD 10) mm Hg between the intervention and control groups to be a clinically relevant difference [57-59]. Therefore, the required number of patients per group was 104, with a 2-tailed α level set at 5% and the statistical power at 95%. Accounting for a 20% dropout rate during follow-up, the sample size was increased to at least 125 patients in each group.

### Patient Randomization and Trial Implementation

Patients were randomized 1:1 to the intervention and control groups based on a computer-generated series of numbers using Excel’s random macro function and sequentially numbered, opaque, and sealed envelope technique [60]. The randomization group was printed on paper and retained in an opaque sealed envelope.

Patients in the intervention group were required to download Blood Pressure Assistant onto their smartphones by scanning a QR code. The nurse then provided the participants with 10 to 15 minutes of training regarding the method to navigate and use the available functional modules of Blood Pressure Assistant and asked them to use it for 6 months.

The control group was managed on the basis of recommendations by the *2018 Chinese Guidelines for Prevention and Treatment of Hypertension* [13], which included telephone follow-ups or outpatient clinic management. Patients from both groups were assessed with a paper-based questionnaire survey and were educated on hypertension knowledge at the baseline and 6 months.

Participants in both groups were required to measure their BP using their own sphygmomanometer and record the results on the app (intervention group) or in a notebook (control group) at home twice a day: 1 recording conducted between 6 and 9 AM, after bladder voiding and before taking antihypertensive medications, and another conducted between 6 and 9 PM, after dinner and before sleeping.

To ensure the accuracy and reliability of these devices, participants in both groups were trained by a nurse in the proper use and maintenance of the BP devices, including proper cuff size and placement; positioning of the arm at the heart level; allowing for a resting period before taking a measurement; and avoiding caffeine, exercise, or stress, before taking a reading. They were also asked to periodically compare readings with those taken during their clinic visit. If discrepancies were noticed, they were requested to recalibrate the sphygmomanometer.

The “health manager” team conducted a follow-up telephone call with participants in both groups at 3 months. For the intervention group, when the system detected abnormal BP readings or patient-reported discomfort and prolonged nonuse of the app (1 month), the web-based portal would automatically send out a warning signal, alerting the team to conduct follow-up phone calls to provide appropriate assistance. Participants in the control group were generally directed to continue their normal activities and follow any medical advice or treatment recommendations they received from the health professionals in the training session. They were also advised to call the management team for advice or to seek medical attention immediately if their home BP readings were too high or too low.

### Primary Outcome

Blood pressure control was measured with the control rate (ie, the proportion of participants with an SBP of <140 mm Hg and DBP of <90 mm Hg) and SBP and DBP readings at baseline and 6 months measured using the regularly calibrated Omron automatic electronic sphygmomanometer (HEM-7052; Omron Dalian Co Ltd) during the clinical visit. When a patient arrived at the hospital, a nurse would ask him or her to fill out the questionnaire first; then measure BP, height, and weight; and finally conduct a blood test. To reduce variation in the readings, all BP measurements were taken using the standardized technique, following the recommended method by the American Heart Association [61]. To prevent bias, the nurses conducting the measurements were blinded to group allocation because they constituted a separate team responsible for group allocation. All analyses included both the complete case and an intention-to-treat (ITT) analysis [62,63]. The ITT analysis included the imputed data for continuous losses in subsequent observations, using the expectation-maximization method and incorporating missing follow-up data through a dichotomous approach for predicting average matching.

### Secondary Outcome

An 8-item questionnaire survey was used to compare the changes in hypertension knowledge in each group and between the 2 groups at baseline and 6 months. The items included (1) definition of hypertension, (2) treatment, (3) risk factors, (4) comorbidities, (5) prevention, (6) salt intake, (7) medication use, and (8) hypertension classification. For each item, the correct answer was scored as 1; otherwise, it was scored as 0. The overall awareness rate of hypertension knowledge was calculated by the proportion of the correct answers to all responses to each question.

A 6-item questionnaire survey was used to compare a patient’s changes in lifestyle behavior at baseline and 6 months. The items included (1) cigarette smoking, (2) alcohol consumption, (3) healthy dietary habit, (4) low-salt intake, (5) physical exercise, and (6) and antihypertensive medication adherence. The options 3 to 5 in each item are equidistantly quantified in accordance with the degree of compliance from high to low. For example, for an item with 3 options, complete compliance was scored as 1, partial compliance was scored as 0.5, and noncompliance was scored as 0. The overall compliance rate of lifestyle behavior was calculated by the average score of all 6 questions.

Blood glucose levels (including normal blood glucose and impaired fasting glucose), blood lipid levels (including low risk, moderate risk, and high risk), waist-hip ratio, and BMI were measured at baseline and 6 months.

### Statistical Analyses

All data were analyzed using SPSS (version 21.0; IBM Corp). Continuous data are expressed as mean and SD values. Comparisons between the 2 groups were carried out using the 2-tailed *t* test or chi-square test. A *P* value of <.05 was considered statistically significant.

## Results

### Participant Characteristics

**Figure 2:**
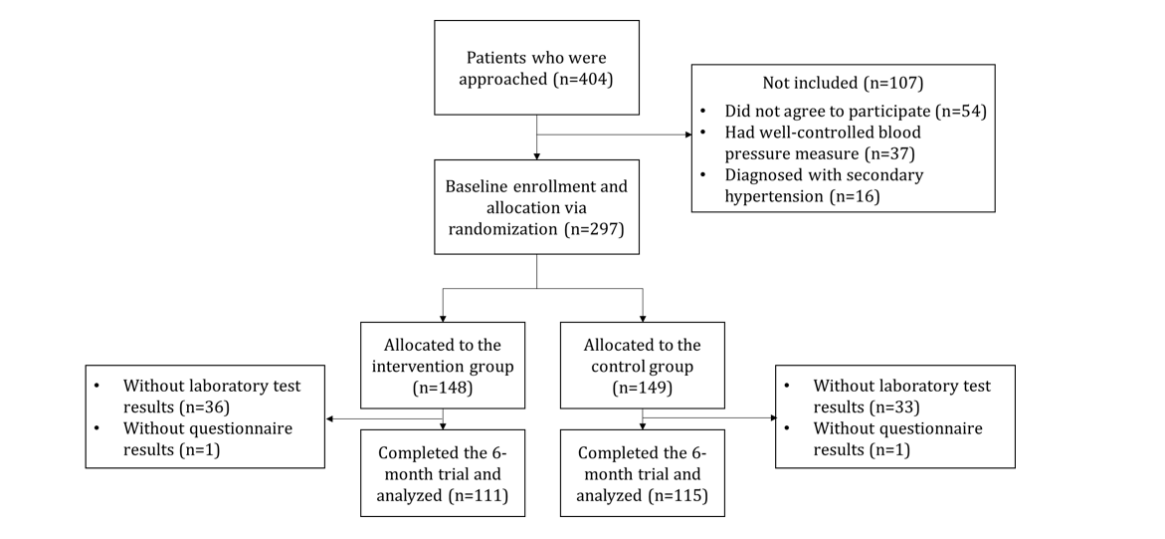
Flow diagram of patient selection and treatment.

Out of 404 patients screened, 297 were included in the study and equally randomized to the intervention and control groups (n=148 vs n=149, respectively). The participants included in the analysis were diverse in age, sex, education level, years of experiencing hypertension, comorbidities, and genetic history, with no significant differences in these demographic characteristics and medication use between the 2 groups (all *P*>.05; Table 1 and Multimedia Appendix 1). In the intervention group, 111 participants completed the 6-month trial, and the attrition rate was 25% (37/148), while in the control group, 115 participants completed the 6-month trial, and the attrition rate was 22.8% (34/149; Figure 2). The lack of laboratory test results was the main reason for the loss to follow-up. We reached out to all patients who dropped off to ask them why they could not complete the final assessment on time. They either replied that they were not available or they did not answer the call. The demographic and clinical characteristics of these dropouts differed from those who remained in the study.

**Table 1:** Baseline participant characteristics.

Characteristics		Intervention group (n=148)	Control group (n=149)	*P* value
Age (years), mean (SD)		48.58 (9.54)	50.64 (8.72)	.09
**Sex, n (%)**				.40
	Male	78 (52.7)	70 (47)	
	Female	70 (47.3)	79 (53)	
**Education level (years), n (%)**				.10
	≥12	62 (41.9)	77 (51.7)	
	<12	86 (58.1)	72 (48.3)	
**Years of experiencing hypertension, n (%)**				.18
	1	54 (36.5)	58 (38.9)	
	1-5	50 (33.8)	39 (26.2)	
	6-10	33 (22.3)	31 (20.8)	
	>10	11 (7.4)	21 (14.1)	
**Comorbidities, n (%)**				.79
	Yes	70 (47.3)	69 (46.3)	
	No	78 (52.7)	80 (53.7)	
**Genetic history, n (%)**				.15
	Yes	115 (77.7)	108 (72.5)	
	No	33 (22.3)	41 (27.5)	

### Intervention Outcomes

**Figure 3:**
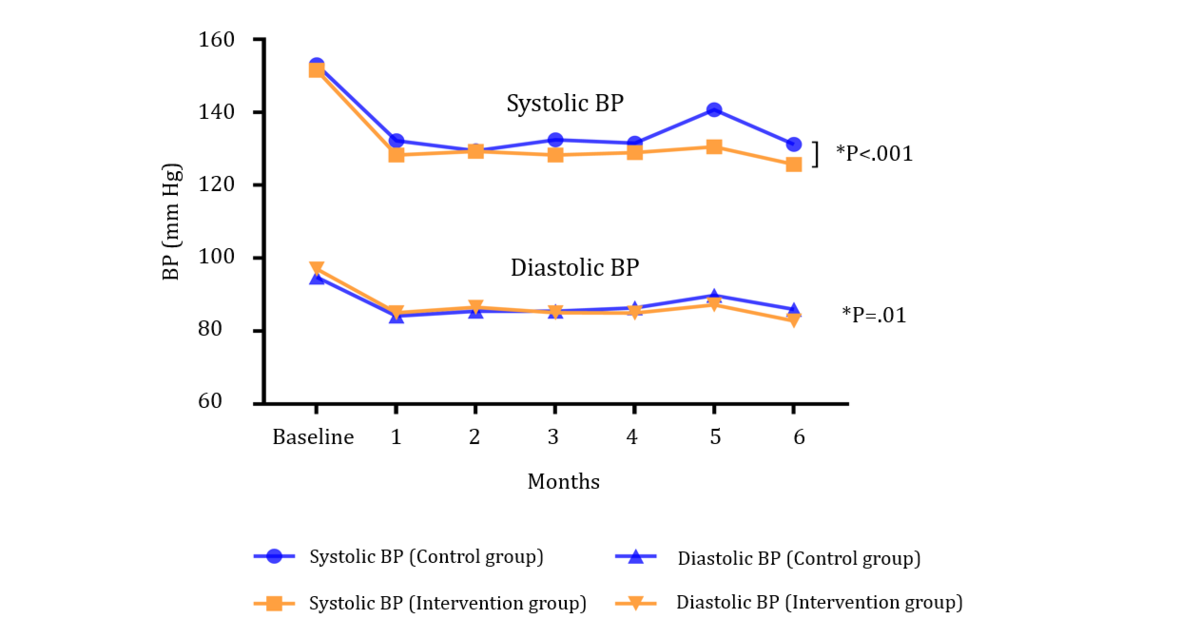
Comparison of blood pressure changes in the two groups. BP: blood pressure.

For all measures, there was no significant difference between the 2 groups at baseline (all *P*>.05; Table 1). The results of the ITT analysis and complete case analysis did not substantially differ (Table 2). BP was not controlled (<140 and <90 mm Hg for SBP and DBP, respectively) for all participants in both groups at baseline. Despite a significantly improved control rate in both groups at 6 months (both *P*<.001), there was a significantly higher control rate in the intervention group (100/111, 90.1%) than in the control group (75/115, 65.2%; *P*<.001). The mean SBP and DBP values in the intervention group were significantly reduced by 25.83 (SD 8.99) and14.28 (SD 3.74) mm Hg at 6 months, respectively; in the control group, a significant reduction by 21.83 (SD 6.86) and 8.87 (SD 4.22) mm Hg was observed, respectively. At 6 months, the differences in SBP and DBP between the 2 groups were significant (*P*<.001 and *P*=.01, respectively; Figure 3).

**Table 2:** Comparison of pre- and postintervention measures between the 2 groups.

Measure			Intervention group	Control group	*P* value
**Complete case analysis (intervention: n=111; control: n=115)**					
	**Blood pressure control rate, n (%)**				
		Before the study	0 (0)	0 (0)	>.99
		After the study	100 (90.1)	75 (65.2)	<.001^a^
		*P* value on pre-post comparison	<.001^a^	<.001^a^	N/A^b^
	**Systolic blood pressure (mm Hg), mean (SD)**				
		Before the study	151.51 (17.38)	153.00 (18.68)	.58
		After the study	125.68 (8.39)	131.17 (11.82)	<.001^a^
		*P* value on pre-post comparison	<.001^a^	<.001^a^	N/A
	**Diastolic blood pressure (mm Hg), mean (SD)**				
		Before the study	97.06 (11.08)	94.81 (13.08)	.22
		After the study	82.78 (7.34)	85.94 (8.86)	.01
		*P* value on pre-post comparison	<.001^a^	<.001^a^	N/A
	**Hypertension knowledge score, mean (SD)**				
		Before the study	5.31 (1.72)	5.15 (1.80)	.54
		After the study	6.93 (1.23)	5.48 (1.86)	<.001^a^
		*P* value on pre-post comparison	<.001^a^	.23	N/A
	**Lifestyle assessment score, mean (SD)**				
		Before the study	3.32 (1.27)	3.61 (1.15)	.11
		After the study	4.12 (1.03)	3.39 (1.15)	.24
		*P* value on pre-post comparison	<.001^a^	.71	N/A
	**BMI (kg/m^2^), mean (SD)**				
		Before the study	25.87 (3.16)	26.74 (3.42)	.08
		After the study	25.36 (2.81)	26.81 (3.47)	.002^a^
		*P* value on pre-post comparison	.25	.89	N/A
**Intention-to-treat analysis (intervention: n=148; control: n=149)**					
	**Blood pressure control rate, n (%)**				
		Before the study	0 (0)	0 (0)	>.99
		After the study	96 (64.9)	73 (49)	<.001^a^
		*P* value on pre-post comparison	<.001^a^	<.001^a^	N/A
	**Systolic blood pressure (mm Hg), mean (SD)**				
		Before the study	151.80 (18.37)	153.79 (19.77)	.40
		After the study	127.42 (19.77)	134.72 (10.75)	<.001^a^
		*P* value on pre-post comparison	<.001^a^	<.001^a^	N/A
	**Diastolic blood pressure (mm Hg), mean (SD)**				
		Before the study	98.59 (10.97)	96.34 (13.39)	.14
		After the study	84.21 (7.64)	87.55 (9.66)	.002^a^
		*P* value on pre-post comparison	<.001^a^	<.001^a^	N/A
	**Hypertension knowledge score, mean (SD)**				
		Before the study	4.50 (1.92)	4.43 (1.95)	.75
		After the study	6.71 (1.42)	5.37 (2.00)	<.001^a^
		*P* value on pre-post comparison	<.001^a^	.23	N/A
	**Lifestyle assessment score, mean (SD)**				
		Before the study	3.29 (1.75)	3.54 (1.24)	.13
		After the study	4.08 (1.16)	3.16 (1.32)	.04^a^
		*P* value on pre-post comparison	<.001^a^	.73	N/A
	**BMI (kg/m^2^), mean (SD)**				
		Before the study	26.17 (3.23)	26.27 (3.67)	.08
		After the study	25.65 (2.98)	26.28 (3.60)	.01^a^
		*P* value on pre-post comparison	.25	.89	N/A

^a^Significant at *P*<.05.

^b^N/A: not applicable.

**Figure 4:**
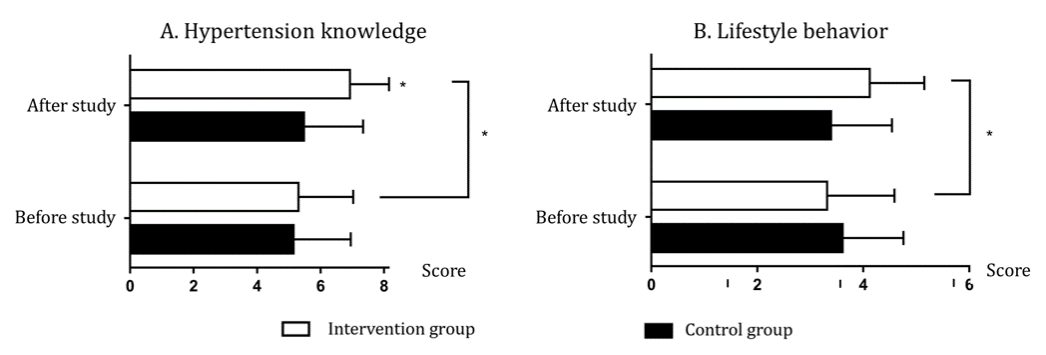
Comparison of hypertension knowledge and lifestyle behavior between the 2 groups. *P<.05.

Hypertension knowledge and lifestyle assessment scores significantly improved in the intervention group at 6 months (all *P*<.001) but not in the control group (all *P*>.05). The differences between the intervention and control groups in hypertension knowledge scores were significant (*P*<.001) but not in lifestyle assessment scores (*P*=.24). The questionnaire’s outcomes suggest that the participants in the intervention group had significantly improved knowledge of the 8 aspects of hypertension and its management (all *P*<.05; Figure 4A and Multimedia Appendix 2). Regarding lifestyle management, the intervention group had significant improvements in medication intake (*P*<.001), healthy diet (*P*=.02), low salt intake (*P*<.001), and physical exercise (*P*=.02), while there was no significant change in smoking (*P*=.50) and alcohol consumption (*P*=.30; Figure 4B and Multimedia Appendix 3).

Although BMI in the 2 groups did not change significantly before and after the study (all *P*>.05), at 6 months, participants in the intervention group had a significantly lower BMI than those in the control group (*P*=.002). No significant differences were found in blood glucose levels, blood lipid levels, and waist-hip ratio in either pre-post or intergroup comparisons at 6 months (all *P*>.05).

## Discussion

### Principal Findings

This study assesses the short-term efficacy of an mHealth app–based intervention to support patient self-management of hypertension in a lower-income region with a high-salt diet in China. We compared the efficacy of an mHealth app–based intervention with that of usual care in supporting patient self-management of hypertension through a 6-month randomized controlled trial with 226 participants. There were no significant differences in demographic characteristics and medication use between the 2 groups of participants. Hence, the significant differences in evaluation outcomes between the 2 groups might be attributed to the efficacy of the mHealth intervention.

Participants in both groups showed a significant reduction in SBP and DBP at 6 months compared to those at baseline. This suggests that traditional treatment methods can effectively help patients to control their BP [64,65]. However, the BP control rate in the intervention group was significantly higher than that in the control group, showing that the mHealth app–based intervention can complement traditional hypertension management and further enhance patients’ self-management behaviors. This is in line with findings from other areas about mHealth’s role in supporting patient self-management, such as diabetes, cancer, asthma, and smoking or alcohol cessation [66-70]. The participants used a home-based ambulatory BP monitoring device to measure BP in the comfort of their homes, which minimized the “white-coat effect” of artificially elevated BP in the clinic. This could be a better strategy for collecting patients’ BP data compared to those used in previous studies [48,49,51,70,71].

It is noteworthy that the control group also experienced a significant improvement in BP outcomes. Three possible reasons can be posited to elucidate this occurrence. First, the research itself may have engendered a placebo effect to some degree. Despite the absence of an mHealth intervention for the control group participants, their BP outcomes might have been inadvertently affected. Second, it is possible that participation in the study heightened their cognizance and emphasis on BP control. Lastly, it is conceivable that some participants in the control group acquired a more sophisticated comprehension of BP management during subsequent clinical visits. Further research is required to control for potential confounding factors to more accurately ascertain the specific effects of the mHealth intervention.

We observed no significant differences in baseline weight between the 2 groups at baseline and after the intervention. However, a significant weight change was observed within the intervention group on comparing pre- and postintervention measures. This alteration could potentially be attributed to the implementation of the app, such as educational material and reminders about diet modifications and physical activities. While the control group did not receive the same intervention via electronic means, they were provided with traditional self-management training, suggesting that traditional management remains effective, with mHealth further enhancing responsiveness and efficiency. The weight change observed in the intervention group might be attributed to the use of the mHealth app. Nevertheless, it remains inconclusive whether the intervention exerted a uniform effect on all participants, implying that interindividual variability could have impacted the overall weight change in this group. The observed results might also be subject to random factors, thus necessitating further studies to determine the generalizability of these findings.

Patients’ awareness of all 8 aspects of hypertension knowledge in the intervention group has significantly improved during both pre-post and intergroup comparisons after the trial. This confirms the efficacy of the mHealth intervention in providing health education to patients [20,72]. A challenge for patients’ self-management of hypertension is that patients often overlook or feel overwhelmed by the arduous process of self-management [73]. The mHealth intervention is helpful in providing patients with access to educational information anytime and anywhere, thereby raising their awareness and improving their ability to self-manage their conditions, thus achieving good BP control [43,52].

Self-reported management behaviors with regard to diet, physical activity, and medication adherence in the intervention group significantly improved 6 months after the intervention compared to those at baseline. However, there was no significant change in cigarette smoking and alcohol consumption, reflecting the considerable challenge of giving up addictive substances [67,74]. A positive change was reducing salt intake, which is a significant transformation from the high-salt traditional diet—a significant risk factor for the high prevalence of hypertension in this region. In addition, medication adherence significantly improved in both groups, which positively contributed to BP control.

Nonetheless, there are potential problems associated with the use of such an app. If many patients use the app, health care providers may be overwhelmed by the workload of manually reviewing and responding to patient data. One solution might be to develop automated processes for data analysis. Thus, follow-up studies could consider using artificial intelligence technologies, such as chatbots and predictive algorithms, to address this issue. Examples include answering frequently asked questions and providing general information to patient users, thus reducing the need for health care providers to respond to such inquiries, and monitoring a patient’s progress over time, identifying potential health issues and allowing health care providers to focus on higher-priority cases. However, it is worth noting that these technologies are still under development and should be validated before being used in clinical practice. Health care providers should validate the predictions made by these algorithms to ensure patient safety.

### Limitations

This study has limitations. First, patient recruitment was conducted using convenience sampling from a single site, which may be biased by demographic characteristics and might limit the generalizability of the results. However, this study lays the groundwork for future research on the efficacy of the mHealth app in a larger sample of patients with hypertension at multiple sites. Second, the study had a relatively high attrition rate. However, the comparison of baseline data of the remaining participants of the 2 groups found no significant difference, and the results are still representative. Follow-up studies are needed to further identify reasons for participant attrition to prevent future dropouts and the incentives that can help keep participants engaged and reduce the likelihood of dropping out. Third, all participants in the intervention group were mobile phone owners and likely to have a higher socioeconomic status. Previous studies have suggested that this population is at a lower risk of nonadherence to medication management [75,76]. In addition, not having a smartphone makes it more difficult for individuals to be recruited for clinical trials and for trials to collect accurate and complete data, particularly by relying on mobile technologies to collect data and track self-management progress. Fourth, hypertension in patients was monitored on the basis of their self-reported BP measurement instead of accurate measurement by health care providers. Although participants were instructed to measure BP more accurately at the same time each day, it was not guaranteed that all of them followed the instructions, which may cause an error in the measurement outcomes. Therefore, follow-up research can collect data such as the app functions used and the frequency of patients using the app to analyze which functions affect BP control among participants. Strategies for assessing the accuracy of patient-uploaded data and how to engage their family members to facilitate self-management among patients who cannot self-manage their condition also need to be further explored. Fifth, as the intervention trial could not be blinded in our study context, it may lead to a tendency for patients in the intervention group to report better BP readings. However, we used a multicomponent intervention (eg, health manager team monitoring) and performed pre- and postcontrol measurements on objective data to maximize the objectivity of the study.

### Conclusions

This study reports that the mHealth app–based intervention for patient self-management of hypertension may be able to improve their health knowledge and enable them to improve self-management toward a healthy lifestyle, including adherence to medication, a low-salt diet, and physical activities, thereby achieving optimal BP control at 6 months after the intervention. The mHealth innovation addresses the gaps for health providers and organizations to support patients with chronic disease self-management by providing better access to educational material, ongoing monitoring, and feedback. Further research is needed to control for potential confounding factors to ascertain the specific effects of these interventions more accurately.

## Appendix

Multimedia Appendix 1 The medication use between the 2 groups.

Multimedia Appendix 2 Self-assessment of hypertension knowledge.

Multimedia Appendix 3 Self-assessment of lifestyle behaviors.

Multimedia Appendix 4 CONSORT-EHEALTH checklist.

## Abbreviations

BP: blood pressure
DBP: diastolic blood pressure
ITT: intention-to-treat
mHealth: mobile health
SBP: systolic blood pressure
