# Supplementary material for: Efficacy of an mHealth App to Support Patients’ Self-Management of Hypertension: Randomized Controlled Trial
Source: J Med Internet Res. 2023 Dec 19;25:e43809. doi: 10.2196/43809 (PMC10762623; doi:10.2196/43809)
Supplement: Multimedia Appendix 1 [file jmir_v25i1e43809_app1.pdf]

Multimedia Appendix 1. The medication use between the two groups.

		Control group	Intervention group	Total
ACEI	Count	7_a	5_a	12
	%	4.7%	3.4%	4.0%
ACEI+BB	Count	1_a	0_a	1
	%	0.7%	0.0%	0.3%
ARB	Count	18_a	15_a	33
	%	12.1%	10.1%	11.1%
ARB+BB	Count	3_a	5_a	8
	%	2.0%	3.4%	2.7%
ARB+Diuretic	Count	4_a	0_b	4
	%	2.7%	0.0%	1.3%
BB	Count	2_a	3_a	5
	%	1.3%	2.0%	1.7%
CCB	Count	26_a	34_a	60
	%	17.4%	23.0%	20.2%
CCB+ACEI	Count	11_a	15_a	26
	%	7.4%	10.1%	8.8%
CCB+ACEI+BB	Count	2_a	6_a	8
	%	1.3%	4.1%	2.7%
CCB+ACEI+BB+Diuretic	Count	1_a	0_a	1
	%	0.7%	0.0%	0.3%
CCB+ACEI+Diuretic	Count	3_a	7_a	10
	%	2.0%	4.7%	3.4%
CCB+ARB	Count	31_a	28_a	59
	%	20.8%	18.9%	19.9%
CCB+ARB+BB	Count	13_a	5_a	18
	%	8.7%	3.4%	6.1%
CCB+ARB+BB+Diuretic	Count	1_a	2_a	3
	%	0.7%	1.4%	1.0%
CCB+ARB+Diuretic	Count	9_a	4_a	13
	%	6.0%	2.7%	4.4%
CCB+BB	Count	16_a	16_a	32
	%	10.7%	10.8%	10.8%
CCB+BB+Diuretic	Count	0_a	1_a	1
	%	0.0%	0.7%	0.3%
CCB+Diuretic	Count	0_a	1_a	1
	%	0.0%	0.7%	0.3%
Diuretic	Count	1_a	1_a	2
	%	0.7%	0.7%	0.7%
Total	Count	149	148	297
	%	100.0%	100.0%	100.0%

Calcium channel blockers (CCB), β blockers (BB), Angiotensin-converting enzyme inhibitors (ACEI), angiotensin receptor blockers (ARB)
