# Supplementary material for: Efficacy of an mHealth App to Support Patients’ Self-Management of Hypertension: Randomized Controlled Trial
Source: J Med Internet Res. 2023 Dec 19;25:e43809. doi: 10.2196/43809 (PMC10762623; doi:10.2196/43809)
Supplement: Multimedia Appendix 2 [file jmir_v25i1e43809_app2.pdf]

Multimedia Appendix 2. Self-assessment of hypertension knowledge

	**Intervention group (n=111)**	**Control group (n=115)**	***P-value***
Question 1 Hypertension Definition			
Before study	0.54±0.50	0.39±0.49	.037
After study	0.79±0.41	0.53±0.50	<.001
P-value, Before-after comparison	<.001	.052	
Question 2 Treatment for Hypertension			
Before study	0.76±0.43	0.73±0.44	.734
After study	0.91±0.29	0.74±0.43	.003
P-value, Before-after comparison	.005	.866	
Question 3 Risk Factors for Hypertension			
Before study	0.72±0.34	0.64±0.32	.094
After study	0.91±0.23	0.68±0.34	<.001
P-value, Before-after comparison	<.001	.403	
Question 4 Hypertension Complications			
Before study	0.76±0.34	0.64±0.42	.034
After study	0.86±-.26	0.60±0.37	<.001
P-value, Before-after comparison	.035	.534	
Question 5 Prevention			
Before study	0.82±0.31	0.78±0.31	.45
After study	0.96±0.14	0.78±0.33	<.001
P-value, Before-after comparison	<.001	.963	
Question 6 Salt Intake			
Before study	0.27±0.44	0.30±0.46	.62
After study	0.66±0.48	0.38±0.48	.001
P-value, Before-after comparison	<.001	.273	
Question 7 Hypertensive Medication			
Before study	0.73±0.44	0.82±0.38	.153
After study	0.91±0.29	0.91±0.29	1.00
P-value, Before-after comparison	.002	.08	
Question 8 Hypertension Classification			
Before study	0.71±0.46	0.84±0.36	.032
After study	0.93±0.25	0.84±0.36	0.058
P-value, Before-after comparison	<.001	1.00	
