# Supplementary material for: Efficacy of an mHealth App to Support Patients’ Self-Management of Hypertension: Randomized Controlled Trial
Source: J Med Internet Res. 2023 Dec 19;25:e43809. doi: 10.2196/43809 (PMC10762623; doi:10.2196/43809)
Supplement: Multimedia Appendix 3 [file jmir_v25i1e43809_app3.pdf]

Multimedia Appendix 3. Self-assessment of lifestyle behaviors

	**Intervention Group (n=111)**	**Control Group (n=115)**	***P-value***
Question 1 Cigarette Smoking			
Before study	0.79±0.35	0.80±0.34	.845
After study	0.83±0.31	0.81±0.33	.783
P-value, Before-after comparison	.499	.842	
Question 2 Alcohol Consumption			
Before study	0.50±0.42	0.63±0.42	0.121
After study	0.57±0.40	0.64±0.42	0.225
P-value, Before-after comparison	.300	.765	
Question 3 Healthy Dietary Habits			
Before study	0.65±0.44	0.63±0.42	.362
After study	0.78±0.36	0.64±0.42	.837
P-value, Before-after comparison	.024	.250	
Question 4 Low-salt Diet			
Before study	0.37±0.32	0.71±0.41	.549
After study	0.53±0.25	0.77±0.37	.299
P-value, Before-after comparison	<.001	.065	
Question 5 Exercise			
Before study	0.41±0.39	0.41±0.41	.911
After study	0.55±0.37	0.36±0.40	.001
P-value, Before-after comparison	.017	.440	
Question 6 Antihypertensive Medication			
Before study	0.41±0.74	0.56±0.67	.165
After study	0.82±0.46	0.82±0.28	.558
P-value, Before-after comparison	<.001	<.001	
