# Supplementary material for: Efficacy of an mHealth App to Support Patients’ Self-Management of Hypertension: Randomized Controlled Trial
Source: J Med Internet Res. 2023 Dec 19;25:e43809. doi: 10.2196/43809 (PMC10762623; doi:10.2196/43809)
Supplement: Multimedia Appendix 4 [file jmir_v25i1e43809_app4.pdf]

**CONSORT-EHEALTH Checklist V1.6.2 Report**	**Manuscript Number**	43809
**(based on CONSORT-EHEALTH V1.6), available at [http://tinyurl.com/consort-ehealth-v1-6].**		
**Date completed**		
11/29/2023 10:01:37		
**by**		
Fang Liu		
Efficacy of an mHealth App to Support Patients' Self-Management of Hypertension: Randomized Controlled Trial		
TITLE		
**1a-i) Identify the mode of delivery in the title**		
Efficacy of an "mHealth" App to Support Patients' Self-Management of Hypertension: Randomized Controlled Trial		
**1a-ii) Non-web-based components or important co-interventions in title**		
Emphasize the study's innovative aspect, as it represents the most novel component. The choice was also driven by the need for brevity and strategic focus in capturing the attention of our target audience.		
**1a-iii) Primary condition or target group in the title**		
Efficacy of an "mHealth" App to Support "Patients"' Self-Management of Hypertension: Randomized Controlled Trial		
ABSTRACT		
**1b-i) Key features/functionalities/components of the intervention and comparator in the METHODS section of the ABSTRACT**		
They could use the app to "record and upload vital signs, access educational materials, and receive self-management reminders and feedback from health care providers based on the analysis of the uploaded data".		
**1b-ii) Level of human involvement in the METHODS section of the ABSTRACT**		
They could use the app to record and upload vital signs, access educational materials, and "receive self-management reminders and feedback from health care providers based on the analysis of the uploaded data".		
**1b-iii) Open vs. closed, web-based (self-assessment) vs. face-to-face assessments in the METHODS section of the ABSTRACT**		
Participants selected via convenience sampling were "randomly allocated into intervention and control groups". Intervention group participants were "trained" and asked to use an mHealth app named Blood Pressure Assistant for 6 months.		
**1b-iv) RESULTS section in abstract must contain use data**		
A 2-arm randomized controlled trial was conducted among "297 patients" with hypertension		
**1b-v) CONCLUSIONS/DISCUSSION in abstract for negative trials**		
The results of our experiment were positive.		
INTRODUCTION		
**2a-i) Problem and the type of system/solution**		
Despite recognizing the harm of hypertension and the efficacy of traditional hypertension treatment approaches, hypertension control remains suboptimal for most patients, even in high-income countries [6,7].<br>mHealth is increasingly considered by decision makers as a potential low-cost solution to automatically link community-dwelling patients with their health care providers and to innovate hypertension outpatient services to address the abovementioned hypertension self-management challenges [31,32].		
**2a-ii) Scientific background, rationale: What is known about the (type of) system**		
"Despite numerous mHealth apps having entered the market to support patients' self-management of hypertension, the quality of these apps varies largely [33-36]. Although some apps were well-designed and can support patients' self-management of hypertension [37-42], overall, the efficacy of mHealth has not been established due to the heterogeneity of studies in terms of a theoretical basis, intervention design, implementation process and duration, and patient characteristics [39,40,43-45]. Moreover, the existent empirical research on the role of mHealth in supporting patients' self-management of hypertension has been mainly conducted in Western countries [46-51]. "		
**Does your paper address CONSORT subitem 2b?**		
"Therefore, this study aims to compare the efficacy of an mHealth app–based intervention with usual care in supporting patients' self-management of hypertension. "		

METHODS		
**3a) CONSORT: Description of trial design (such as parallel, factorial) including allocation ratio**		
A 2-arm, parallel, prospective randomized controlled trial (retrospectively registered in the Chinese Clinical Trial Registry: ChiCTR1900026437) was conducted to assess the 6-month effects of an mHealth app–based intervention for patients' self-management of hypertension. Patients were randomized 1:1 to the intervention and control groups based on a computer-generated series of numbers using Excel's random macro function and sequentially numbered, opaque, and sealed envelope technique [60].		
**3b) CONSORT: Important changes to methods after trial commencement (such as eligibility criteria), with reasons**		
Our approach did not change significantly after the start of the trial.		
**3b-i) Bug fixes, Downtimes, Content Changes**		
Our app was not upgraded during the trial process and the content was consistent.		
**4a) CONSORT: Eligibility criteria for participants**		
"Inclusion criteria were (1) being aged 18 to 80 years; (2) being diagnosed with primary hypertension, defined as a systolic BP (SBP) of ≥140 mm Hg or diastolic BP (DBP) of ≥90 mm Hg [13]; (3) receiving antihypertensive medication treatment for over 1 month; and (4) owning a smartphone and being able to use it. Exclusion criteria were (1) being diagnosed with secondary hypertension, (2) being unable to express their perceptions because of mental disabilities or an inability to speak, and (3) not having a sphygmomanometer at home. "		
**4a-i) Computer / Internet literacy**		
"and (4) owning a smartphone and being able to use it. "		
**4a-ii) Open vs. closed, web-based vs. face-to-face assessments:**		
"Potential participants were contacted by a registered nurse during their clinic visit. After each person's visit, the registered nurse talked with them for about 5 minutes, informed them of the purpose and detailed procedure of the study, and sought their consent to participate."		
**4a-iii) Information giving during recruitment**		
"Potential participants were contacted by a registered nurse during their clinic visit. After each person's visit, the registered nurse talked with them for about 5 minutes, informed them of the purpose and detailed procedure of the study, and sought their consent to participate."		
**4b) CONSORT: Settings and locations where the data were collected**		
"Participants in both groups were required to measure their BP using their own sphygmomanometer and record the results on the app (intervention group) or in a notebook (control group) at home twice a day: 1 recording conducted between 6 and 9 AM, after bladder voiding and before taking antihypertensive medications, and another conducted between 6 and 9 PM, after dinner and before sleeping."		
**4b-i) Report if outcomes were (self-)assessed through online questionnaires**		
"An 8-item questionnaire survey was used to compare the changes in hypertension knowledge in each group and between the 2 groups at baseline and 6 months. The items included (1) definition of hypertension, (2) treatment, (3) risk factors, (4) comorbidities, (5) prevention, (6) salt intake, (7) medication use, and (8) hypertension classification. For each item, the correct answer was scored as 1; otherwise, it was scored as 0. The overall awareness rate of hypertension knowledge was calculated by the proportion of the correct answers to all responses to each question.<br>A 6-item questionnaire survey was used to compare a patient's changes in lifestyle behavior at baseline and 6 months. The items included (1) cigarette smoking, (2) alcohol consumption, (3) healthy dietary habit, (4) low-salt intake, (5) physical exercise, and (6) and antihypertensive medication adherence. The options 3 to 5 in each item are equidistantly quantified in accordance with the degree of compliance from high to low. For example, for an item with 3 options, complete compliance was scored as 1, partial compliance was scored as 0.5, and noncompliance was scored as 0. The overall compliance rate of lifestyle behavior was calculated by the average score of all 6 questions.<br>"		
**4b-ii) Report how institutional affiliations are displayed**		
Not mentioned in our paper.		
**5) CONSORT: Describe the interventions for each group with sufficient details to allow replication, including how and when they were actually administered**		
**5-i) Mention names, credential, affiliations of the developers, sponsors, and owners**		

"To ensure the accuracy and reliability of these devices, participants in both groups were trained by a nurse in the proper use and maintenance of the BP devices, including proper cuff size and placement; positioning of the arm at the heart level; allowing for a resting period before taking a measurement; and avoiding caffeine, exercise, or stress, before taking a reading. They were also asked to periodically compare readings with those taken during their clinic visit. If discrepancies were noticed, they were requested to recalibrate the sphygmomanometer.<br>The "health manager" team conducted a follow-up telephone call with participants in both groups at 3 months. For the intervention group, when the system detected abnormal BP readings or patient-reported discomfort and prolonged nonuse of the app (1 month), the web-based portal would automatically send out a warning signal, alerting the team to conduct follow-up phone calls to provide appropriate assistance. Participants in the control group were generally directed to continue their normal activities and follow any medical advice or treatment recommendations they receive from the health professionals in the training session. They were also advised to call the management team for advice or to seek medical attention immediately if their home BP readings were too high or too low.<br>"		
**5-ii) Describe the history/development process**		
"The mHealth app–based intervention contains three components: (1) a mobile app named Blood Pressure Assistant, (2) nurse training on the use of the app, and (3) an accredited "health manager" team that follows up with the patient users.<br>Blood Pressure Assistant was codeveloped by the General Hospital of Ningxia Medical University and Zhejiang University to support patients' self-management of hypertension in Ningxia, China, where there is a high prevalence of hypertension due to a high-salt diet [54]. The development of the app was based on the social-cognitive theory, the user acceptance model, and goal-directed design [37,55].<br>"		
**5-iii) Revisions and updating**		
the development and/or content was "frozen" during the trial		
**5-iv) Quality assurance methods**		
The development of the app was based on the social-cognitive theory, the user acceptance model, and goal-directed design [37,55].An accredited "health manager" team, including a senior medical specialist and a senior nurse, were responsible for analyzing these records; monitoring patient conditions; and providing feedback via phone calls, SMS text messages, emails, or social media (eg, WeChat) when deemed necessary.		
**5-v) Ensure replicability by publishing the source code, and/or providing screenshots/screen-capture video, and/or providing flowcharts of the algorithms used**		
To replicate this randomized controlled trial (RCT) comparing hypertension management with and without an app, future researchers should recruit two distinct patient groups with similar baseline characteristics. They must ensure one group uses the designated app for hypertension management, while the other follows standard care without app assistance.		
**5-vi) Digital preservation**		
https://apps.apple.com/us/app/id1100697683		
**5-vii) Access**		
"Patients could self-manage hypertension using the 6 interactive functional modules in Blood Pressure Assistant: health education, health management plan, health checkup, health report, reminders to perform self-management behaviors, and performance ranking. Patients recorded BP readings and other self-management data in the app, including BP and heart rate, medication type and dose, weight, diet, salt intake, physical exercise, and uncomfortable symptoms. Digital questionnaire surveys were also available to assess patient awareness of hypertension, self-management behaviors, and user satisfaction (Figure 1). Details of the app's functions are reported in previous studies [20,37]. There was also a web-based portal for health care providers to monitor and communicate with their patients. All uploaded data from the app can be transferred to the web portal as dynamic electronic health records. Patients in the intervention group were required to download Blood Pressure Assistant onto their smartphones by scanning a QR code. "		
**5-viii) Mode of delivery, features/functionalities/components of the intervention and comparator, and the theoretical framework**		
"Patients in the intervention group were required to download Blood Pressure Assistant onto their smartphones by scanning a QR code. The nurse then provided the participants with 10 to 15 minutes of training regarding the method to navigate and use the available functional modules of Blood Pressure Assistant and asked them to use it for 6 months.<br>The control group was managed on the basis of recommendations by the 2018 Chinese Guidelines for Prevention and Treatment of Hypertension [13], which included telephone follow-ups or outpatient clinic management. Patients from both groups were assessed with a paper-based questionnaire survey and were educated on hypertension knowledge at the baseline and 6 months."		
**5-ix) Describe use parameters**		
"Participants in both groups were required to measure their BP using their own sphygmomanometer and record the results on the app (intervention group) or in a notebook (control group) at home twice a day: 1 recording conducted between 6 and 9 AM, after bladder voiding and before taking antihypertensive medications, and another conducted between 6 and 9 PM, after dinner and before sleeping."		

**5-x) Clarify the level of human involvement**		
“To ensure the accuracy and reliability of these devices, participants in both groups were trained by a nurse in the proper use and maintenance of the BP devices, including proper cuff size and placement; positioning of the arm at the heart level; allowing for a resting period before taking a measurement; and avoiding caffeine, exercise, or stress, before taking a reading. They were also asked to periodically compare readings with those taken during their clinic visit. If discrepancies were noticed, they were requested to recalibrate the sphygmomanometer.”		
**5-xi) Report any prompts/reminders used**		
“All uploaded data from the app can be transferred to the web portal as dynamic electronic health records. An accredited “health manager” team, including a senior medical specialist and a senior nurse, were responsible for analyzing these records; monitoring patient conditions; and providing feedback via phone calls, SMS text messages, emails, or social media (eg, WeChat) when deemed necessary.”		
**5-xii) Describe any co-interventions (incl. training/support)**		
“To ensure the accuracy and reliability of these devices, participants in both groups were trained by a nurse in the proper use and maintenance of the BP devices, including proper cuff size and placement; positioning of the arm at the heart level; allowing for a resting period before taking a measurement; and avoiding caffeine, exercise, or stress, before taking a reading. They were also asked to periodically compare readings with those taken during their clinic visit. If discrepancies were noticed, they were requested to recalibrate the sphygmomanometer.<br>The “health manager” team conducted a follow-up telephone call with participants in both groups at 3 months. For the intervention group, when the system detected abnormal BP readings or patient-reported discomfort and prolonged nonuse of the app (1 month), the web-based portal would automatically send out a warning signal, alerting the team to conduct follow-up phone calls to provide appropriate assistance. ”		
**6a) CONSORT: Completely defined pre-specified primary and secondary outcome measures, including how and when they were assessed**		
“Primary Outcome<br>Blood pressure control was measured with the control rate (ie, the proportion of participants with an SBP of <140 mm Hg and DBP of <90 mm Hg) and SBP and DBP readings at baseline and 6 months measured using the regularly calibrated Omron automatic electronic sphygmomanometer (HEM-7052; Omron Dalian Co Ltd) during the clinical visit. When a patient arrived at the hospital, a nurse would ask him or her to fill out the questionnaire first; then measure BP, height, and weight; and finally conduct a blood test.<br>Secondary Outcome<br>An 8-item questionnaire survey was used to compare the changes in hypertension knowledge in each group and between the 2 groups at baseline and 6 months. The items included (1) definition of hypertension, (2) treatment, (3) risk factors, (4) comorbidities, (5) prevention, (6) salt intake, (7) medication use, and (8) hypertension classification. For each item, the correct answer was scored as 1; otherwise, it was scored as 0. The overall awareness rate of hypertension knowledge was calculated by the proportion of the correct answers to all responses to each question.<br>A 6-item questionnaire survey was used to compare a patient’s changes in lifestyle behavior at baseline and 6 months. The items included (1) cigarette smoking, (2) alcohol consumption, (3) healthy dietary habit, (4) low-salt intake, (5) physical exercise, and (6) and antihypertensive medication adherence. ”		
**6a-i) Online questionnaires: describe if they were validated for online use and apply CHERRIES items to describe how the questionnaires were designed/deployed**		
“Digital questionnaire surveys were also available to assess patient awareness of hypertension, self-management behaviors, and user satisfaction (Figure 1). Details of the app’s functions are reported in previous studies [20,37]. There was also a web-based portal for health care providers to monitor and communicate with their patients. All uploaded data from the app can be transferred to the web portal as dynamic electronic health records. An accredited “health manager” team, including a senior medical specialist and a senior nurse, were responsible for analyzing these records; monitoring patient conditions; and providing feedback via phone calls, SMS text messages, emails, or social media (eg, WeChat) when deemed necessary.”		
**6a-ii) Describe whether and how “use” (including intensity of use/dosage) was defined/measured/monitored**		
“Participants in both groups were required to measure their BP using their own sphygmomanometer and record the results on the app (intervention group) or in a notebook (control group) at home twice a day: 1 recording conducted between 6 and 9 AM, after bladder voiding and before taking antihypertensive medications, and another conducted between 6 and 9 PM, after dinner and before sleeping.”		
**6a-iii) Describe whether, how, and when qualitative feedback from participants was obtained**		
Patients from both groups were assessed with a paper-based questionnaire survey and were educated on hypertension knowledge at the baseline and 6 months.		
**6b) CONSORT: Any changes to trial outcomes after the trial commenced, with reasons**		
"Participants in both groups were required to measure their BP using their own sphygmomanometer and record the results on the app (intervention group) or in a notebook (control group) at home twice a day: 1 recording conducted between 6 and 9 AM, after bladder voiding and before taking antihypertensive medications, and another conducted between 6 and 9 PM, after dinner and before sleeping."		
**7a) CONSORT: How sample size was determined**		
**7a-i) Describe whether and how expected attrition was taken into account when calculating the sample size**		
“Accounting for a 20% dropout rate during follow-up, the sample size was increased to at least 125 patients in each group.”		

**7b) CONSORT: When applicable, explanation of any interim analyses and stopping guidelines**		
“Primary Outcome<br>Blood pressure control was measured with the control rate (ie, the proportion of participants with an SBP of <140 mm Hg and DBP of <90 mm Hg) and SBP and DBP readings at baseline and 6 months measured using the regularly calibrated Omron automatic electronic sphygmomanometer (HEM-7052; Omron Dalian Co Ltd) during the clinical visit. When a patient arrived at the hospital, a nurse would ask him or her to fill out the questionnaire first; then measure BP, height, and weight; and finally conduct a blood test.<br>Secondary Outcome<br>An 8-item questionnaire survey was used to compare the changes in hypertension knowledge in each group and between the 2 groups at baseline and 6 months. The items included (1) definition of hypertension, (2) treatment, (3) risk factors, (4) comorbidities, (5) prevention, (6) salt intake, (7) medication use, and (8) hypertension classification. For each item, the correct answer was scored as 1; otherwise, it was scored as 0. The overall awareness rate of hypertension knowledge was calculated by the proportion of the correct answers to all responses to each question.<br>A 6-item questionnaire survey was used to compare a patient’s changes in lifestyle behavior at baseline and 6 months. The items included (1) cigarette smoking, (2) alcohol consumption, (3) healthy dietary habit, (4) low-salt intake, (5) physical exercise, and (6) and antihypertensive medication adherence. ”		
**8a) CONSORT: Method used to generate the random allocation sequence**		
“Patients were randomized 1:1 to the intervention and control groups based on a computer-generated series of numbers using Excel’s random macro function and sequentially numbered, opaque, and sealed envelope technique [60]. The randomization group was printed on paper and retained in an opaque sealed envelope.”		
**8b) CONSORT: Type of randomisation; details of any restriction (such as blocking and block size)**		
“Patients were randomized 1:1 to the intervention and control groups based on a computer-generated series of numbers using Excel’s random macro function and sequentially numbered, opaque, and sealed envelope technique [60]. The randomization group was printed on paper and retained in an opaque sealed envelope.”		
**9) CONSORT: Mechanism used to implement the random allocation sequence (such as sequentially numbered containers), describing any steps taken to conceal the sequence until interventions were assigned**		
Patients were randomized 1:1 to the intervention and control groups based on a computer-generated series of numbers using Excel’s random macro function and sequentially numbered, opaque, and sealed envelope technique [60]. The randomization group was printed on paper and retained in an “opaque sealed envelope”.		
**10) CONSORT: Who generated the random allocation sequence, who enrolled participants, and who assigned participants to interventions**		
“Patients were randomized 1:1 to the intervention and control groups based on a computer-generated series of numbers using Excel’s random macro function and sequentially numbered, opaque, and sealed envelope technique [60]. The randomization group was printed on paper and retained in an opaque sealed envelope.”		
**11a) CONSORT: Blinding - If done, who was blinded after assignment to interventions (for example, participants, care providers, those assessing outcomes) and how**		
**11a-i) Specify who was blinded, and who wasn’t**		
Due to the nature of our intervention experiment, a double-blind design was not feasible. This is often the case in studies where interventions are clearly visible or experientially different for participants, such as the use of an app for managing hypertension, making it impossible for both the participants and the researchers to be blind to the intervention group assignments.		
**11a-ii) Discuss e.g., whether participants knew which intervention was the “intervention of interest” and which one was the “comparator”**		
Due to the nature of our intervention experiment, a double-blind design was not feasible. This is often the case in studies where interventions are clearly visible or experientially different for participants, such as the use of an app for managing hypertension, making it impossible for both the participants and the researchers to be blind to the intervention group assignments.		
**11b) CONSORT: If relevant, description of the similarity of interventions**		
“Participants in both groups were required to measure their BP using their own sphygmomanometer and record the results on the app (intervention group) or in a notebook (control group) at home twice a day: 1 recording conducted between 6 and 9 AM, after bladder voiding and before taking antihypertensive medications, and another conducted between 6 and 9 PM, after dinner and before sleeping.”		
**12a) CONSORT: Statistical methods used to compare groups for primary and secondary outcomes**		
“All data were analyzed using SPSS (version 21.0; IBM Corp). Continuous data are expressed as mean and SD values. Comparisons between the 2 groups were carried out using the t test or chi-square test. A P value of <.05 was considered statistically significant.”		
**12a-i) Imputation techniques to deal with attrition / missing values**		
As the questionnaire was conducted under the guidance of the researcher, there were no missing values.		
**12b) CONSORT: Methods for additional analyses, such as subgroup analyses and adjusted analyses**		

“An 8-item questionnaire survey was used to compare the changes in hypertension knowledge in each group and between the 2 groups at baseline and 6 months. The items included (1) definition of hypertension, (2) treatment, (3) risk factors, (4) comorbidities, (5) prevention, (6) salt intake, (7) medication use, and (8) hypertension classification. For each item, the correct answer was scored as 1;, otherwise, it was scored as 0. The overall awareness rate of hypertension knowledge was calculated by the proportion of the correct answers to all responses to each question.<br>A 6-item questionnaire survey was used to compare a patient''s changes in lifestyle behavior at baseline and 6 months. The items included (1) cigarette smoking, (2) alcohol consumption, (3) healthy dietary habit, (4) low-salt intake, (5) physical exercise, and (6) and antihypertensive medication adherence. The options 3 to -5 in each item are equidistantly quantified in accordance with the degree of compliance from high to low. For example, for an item with 3 options, complete compliance was scored as 1, partial compliance was scored as 0.5, and noncompliance was scored as 0. The overall compliance rate of lifestyle behavior was calculated by the average score of all 6 questions.<br>”		
RESULTS		
**13a) CONSORT: For each group, the numbers of participants who were randomly assigned, received intended treatment, and were analysed for the primary outcome**		
“Out of 404 patients screened, 297 patients were included in the study and equally randomized to the intervention and control groups (n=148 vs n=149, respectively). The participants included in the analysis were diverse in age, sex, education level, years of experiencing hypertension, comorbidities, and genetic history, with no significant differences in these demographic characteristics and medication use between the 2 groups (all P>.05; Table 1 and Multimedia Appendix 1). In the intervention group, 111 participants completed the 6-month trial, and the attrition rate was 25.0% (37/148), while in the control group, 115 participants completed the 6-month trial, and the attrition rate was 22.89% (34/149; Figure 2). ”		
**13b) CONSORT: For each group, losses and exclusions after randomisation, together with reasons**		
"The lack of laboratory test results was the main reason for the loss to follow-up. We reached out to all patients who dropped off to ask them why they could not complete the final assessment on time. They either replied that they were not available or they did not answer the call. The demographic and clinical characteristics of these dropouts differed from those who remained in the study."		
**13b-i) Attrition diagram**		
“In the intervention group, 111 participants completed the 6-month trial, and the attrition rate was 25.0% (37/148), while in the control group, 115 participants completed the 6-month trial, and the attrition rate was 22.89% (34/149; Figure 2). ”		
**14a) CONSORT: Dates defining the periods of recruitment and follow-up**		
“Participants were recruited from April 2017 through January 2019 at the General Hospital of Ningxia Medical University, Yinchuan, China—a unique tertiary hospital in the province.”		
**14a-i) Indicate if critical “secular events” fell into the study period**		
“The lack of laboratory test results was the main reason for the loss to follow-up. We reached out to all patients who dropped off to ask them why they could not complete the final assessment on time. They either replied that they were not available, or they did not answer the call. The demographic and clinical characteristics of these dropouts differed from those who remained in the study. ”		
**14b) CONSORT: Why the trial ended or was stopped (early)**		
The trial reached its pre-specified endpoint, successfully completing the study period.		
**15) CONSORT: A table showing baseline demographic and clinical characteristics for each group**		
“For all measures, there was no significant difference between the 2 groups at baseline (all P>.05; Table 1). The results of the ITT analysis and complete case analysis did not substantially differ (Table 2). ”		
**15-i) Report demographics associated with digital divide issues**		
“The participants included in the analysis were diverse in age, sex, education level, years of experiencing hypertension, comorbidities, and genetic history, with no significant differences in these demographic characteristics and medication use between the 2 groups (all P>.05; Table 1 and Multimedia Appendix 1). ”		
**16a) CONSORT: For each group, number of participants (denominator) included in each analysis and whether the analysis was by original assigned groups**		
**16-i) Report multiple “denominators” and provide definitions**		

“For all measures, there was no significant difference between the 2 groups at baseline (all P>.05; Table 1). The results of the ITT analysis and complete case analysis did not substantially differ (Table 2). BP was not controlled (<140 and <90 mm Hg for SBP and DBP, respectively) for all participants in both groups at baseline. Despite a significantly improved control rate in both groups at 6 months (both P<.001), there was a significantly higher control rate in the intervention group (90.0%) than in the control group (65.6%; P<.001). The mean SBP and DBP values in the intervention group was significantly reduced by 25.83 (SD 8.99) and14.28 (SD 3.74) mm Hg at 6 months, respectively; in the control group, a significant reduction by 21.83 (SD 6.86) and 8.87 (SD 4.22) mm Hg was observed, respectively. At 6 months, the differences in SBP and DBP between the 2 groups were significant (P<.001 and P=.01, respectively; Figure 3).”		
**16-ii) Primary analysis should be intent-to-treat**		
“For all measures, there was no significant difference between the 2 groups at baseline (all P>.05; Table 1). The results of the ITT analysis and complete case analysis did not substantially differ (Table 2). ”		
**17a) CONSORT: For each primary and secondary outcome, results for each group, and the estimated effect size and its precision (such as 95% confidence interval)**		
“Hypertension knowledge and lifestyle assessment scores significantly improved in the intervention group at 6 months (all P<.001) but not in the control group (all P>.05). The differences between the intervention and control groups in hypertension knowledge scores were significant (P<.001) but not in lifestyle assessment scores (P=.24). The questionnaire’s outcomes suggest that the participants in the intervention group had significantly improved knowledge of the 8 aspects of hypertension and its management (all P<.05; Figure 4A and Multimedia Appendix 2). Regarding lifestyle management, the intervention group had significant improvements in medication intake (P<.001), healthy diet (P=.02), low salt intake (P<.001), and physical exercise (P=.02), while there was no significant change in smoking (P=.50) and alcohol consumption (P=.30; Figure 4B and Multimedia Appendix 3).”		
**17a-i) Presentation of process outcomes such as metrics of use and intensity of use**		
In our study, we primarily focused on measuring attrition rates as the key process outcome, providing insights into participant retention and engagement with the hypertension management app. However, we did not extend our process outcome measures to include other metrics such as average session length or detailed use intensity.		
**17b) CONSORT: For binary outcomes, presentation of both absolute and relative effect sizes is recommended**		
Our study does not involve binary outcomes; therefore, the recommendation to present both absolute and relative effect sizes is not applicable in our context.		
**18) CONSORT: Results of any other analyses performed, including subgroup analyses and adjusted analyses, distinguishing pre-specified from exploratory**		
“Hypertension knowledge and lifestyle assessment scores significantly improved in the intervention group at 6 months (all P<.001) but not in the control group (all P>.05). The differences between the intervention and control groups in hypertension knowledge scores were significant (P<.001) but not in lifestyle assessment scores (P=.24). The questionnaire’s outcomes suggest that the participants in the intervention group had significantly improved knowledge of the 8 aspects of hypertension and its management (all P<.05; Figure 4A and Multimedia Appendix 2). Regarding lifestyle management, the intervention group had significant improvements in medication intake (P<.001), healthy diet (P=.02), low salt intake (P<.001), and physical exercise (P=.02), while there was no significant change in smoking (P=.50) and alcohol consumption (P=.30; Figure 4B and Multimedia Appendix 3).‘		
**18-i) Subgroup analysis of comparing only users**		
We did not do this so this is not applicable.		
**19) CONSORT: All important harms or unintended effects in each group**		
We did not encounter this so this is not applicable.		
**19-i) Include privacy breaches, technical problems**		
We did not encounter this so this is not applicable.		
**19-ii) Include qualitative feedback from participants or observations from staff/researchers**		
We did not do this so this is not applicable.		
DISCUSSION		
**20) CONSORT: Trial limitations, addressing sources of potential bias, imprecision, multiplicity of analyses**		
**20-i) Typical limitations in ehealth trials**		
“This study has limitations. ”		
**21) CONSORT: Generalisability (external validity, applicability) of the trial findings**		
**21-i) Generalizability to other populations**		
“First, patient recruitment was conducted using convenience sampling from a single site, which may be biased by demographic characteristics and might limit the generalizability of the results. ”		

**21-ii) Discuss if there were elements in the RCT that would be different in a routine application setting**		
“hypertension in patients was monitored on the basis of their self-reported BP measurement instead of accurate measurement by health care providers. Although participants were instructed to measure BP more accurately at the same time each day, it was not guaranteed that all of them followed the instructions, which may cause an error in the measurement outcomes.”		
**22) CONSORT: Interpretation consistent with results, balancing benefits and harms, and considering other relevant evidence**		
**22-i) Restate study questions and summarize the answers suggested by the data, starting with primary outcomes and process outcomes (use)**		
”This study assesses the short-term efficacy of an mHealth app–based intervention to support patient self-management of hypertension in a lower-income region with a high-salt diet in China. We compared the efficacy of an mHealth app–based intervention with that of usual care in supporting patient self-management of hypertension through a 6-month randomized controlled trial with 226 participants.<br>Participants in both groups showed a significant reduction in SBP and DBP at 6 months compared to those at baseline. This suggests that traditional treatment methods can effectively help patients to control their BP [64,65]. ”		
**22-ii) Highlight unanswered new questions, suggest future research**		
“Health care providers should validate the predictions made by these algorithms to ensure patient safety.”		
Other information		
**23) CONSORT: Registration number and name of trial registry**		
Chinese Clinical Trial Registry ChiCTR1900026437		
**24) CONSORT: Where the full trial protocol can be accessed, if available**		
There is no full trial protocal.		
**25) CONSORT: Sources of funding and other support (such as supply of drugs), role of funders**		
This project was supported by the 2016 Ningxia Science and Technology Support Project (2016KJHM62); the 2017 Key Research and Development Plan of Ningxia Hui Autonomous Region (2017BY038); and the Natural Science Foundation of Shanghai, China (21ZR1412700).		
**X26-i) Comment on ethics committee approval**		
“This study was approved by the Human Research Ethics Committee of the General Hospital of Ningxia Medical University (ID2018-325). All participants provided formal written informed consent, which the committee approved. ”		
**x26-ii) Outline informed consent procedures**		
“All participants provided formal written informed consent, which the committee approved. ”		
**X26-iii) Safety and security procedures**		
“All names were replaced with codes during electronic data entry to preserve the anonymity and privacy of the participants. The questionnaires were locked in a filing cabinet at the trial hospital. The data were analyzed in an Excel (Microsoft Corp) spreadsheet and stored in a password-protected secure office computer of a researcher. Data security was guarded by firewalls and other security mechanisms imposed by the networked computers at the trial hospital.”		
**X27-i) State the relation of the study team towards the system being evaluated**		
There is no conflict of interest.		
